# Multielectrode Recordings From Identified Neurons Involved in Visually Elicited Escape Behavior

**DOI:** 10.3389/fnbeh.2020.592309

**Published:** 2020-11-09

**Authors:** Alejandro Cámera, Mariano Andres Belluscio, Daniel Tomsic

**Affiliations:** ^1^Instituto de Fisiología, Biología Molecular y Neurociencias (IFIBYNE), UBA-CONICET, Buenos Aires, Argentina; ^2^Instituto de Fisiología y Biofísica Bernardo Houssay, National Council for Scientific and Technical Research (CONICET), Buenos Aires, Argentina; ^3^Facultad de Medicina, Universidad de Buenos Aires, Buenos Aires, Argentina; ^4^Departamento de Fisiología, Biología Molecular y Celular Dr. Héctor Maldonado, Facultad de Ciencias Exactas y Naturales, Universidad de Buenos Aires, Buenos Aires, Argentina

**Keywords:** simultaneous extracellular recording, tetrodes, motion detection, avoidance, crustacean, insect, giant neurons

## Abstract

A major challenge in current neuroscience is to understand the concerted functioning of distinct neurons involved in a particular behavior. This goal first requires achieving an adequate characterization of the behavior as well as an identification of the key neuronal elements associated with that action. Such conditions have been considerably attained for the escape response to visual stimuli in the crab *Neohelice*. During the last two decades a combination of *in vivo* intracellular recordings and staining with behavioral experiments and modeling, led us to postulate that a microcircuit formed by four classes of identified lobula giant (LG) neurons operates as a decision-making node for several important visually-guided components of the crab’s escape behavior. However, these studies were done by recording LG neurons individually. To investigate the combined operations performed by the group of LG neurons, we began to use multielectrode recordings. Here we describe the methodology and show results of simultaneously recorded activity from different lobula elements. The different LG classes can be distinguished by their differential responses to particular visual stimuli. By comparing the response profiles of extracellular recorded units with intracellular recorded responses to the same stimuli, two of the four LG classes could be faithfully recognized. Additionally, we recorded units with stimulus preferences different from those exhibited by the LG neurons. Among these, we found units sensitive to optic flow with marked directional preference. Units classified within a single group according to their response profiles exhibited similar spike waveforms and similar auto-correlograms, but which, on the other hand, differed from those of groups with different response profiles. Additionally, cross-correlograms revealed excitatory as well as inhibitory relationships between recognizable units. Thus, the extracellular multielectrode methodology allowed us to stably record from previously identified neurons as well as from undescribed elements of the brain of the crab. Moreover, simultaneous multiunit recording allowed beginning to disclose the connections between central elements of the visual circuits. This work provides an entry point into studying the neural networks underlying the control of visually guided behaviors in the crab brain.

## Introduction

To fulfill its biological function the escape response to an impending threat needs to be executed quickly. This implies that sensory information about danger stimuli must be transformed into avoidance actions with the shortest delay, a purpose that is favorably achieved by large neurons capable of conveying information in terms of action potentials (Herberholz and Marquart, 2012). The origin of the action potential is thought to be related to the high speed of conduction required to effectively evade predator attacks (Monk and Paulin, 2014).

Electrophysiology remains the dominant methodology to investigate neuronal activity in the range of high temporal resolution (milliseconds) that characterizes the transfer of information within the nervous system. Electrophysiological measurements can be achieved by intracellular or extracellular recordings. Intracellular recording with sharp electrodes or whole-cell patch provides very detailed data on neurons (i.e., sub-threshold activity) and allows one to make a morphological identification of the recorded neuron. However, it is usually limited to one cell at a time, requires movement restriction, and can be sustained for a relatively short time. On the other hand, the extracellular recording is more easily performed and can be maintained for hours, but only brings information about action potential activity, without direct knowledge of which neuron originated the recorded spike firing. Therefore, these two techniques bring about complementary information.

In part due to the presence of very large neurons involved in avoidance responses, invertebrates have been suitable models to investigate the neuronal physiology using intracellular recordings (e.g., Kandel, 1976; Edwards et al., 1999; Fotowat and Gabbiani, 2011). In these models, the study of neuronal circuit activity has been mostly satisfied by pooling single-cell data from different individuals. This introduces two types of variability, inter-individual and trial-to-trial variability. Therefore, to analyze information encoded in the activity of neuronal populations it is more appropriate to record the activity of several neurons at the same time in the same individual. With multi-channel electrodes and spike sorting fairly large populations of neurons can be analyzed simultaneously (Gray et al., 1995; Buzsáki, 2004; Brill et al., 2013; Rossant et al., 2016). Indeed, research on invertebrates considering groups or populations of neurons instead of single neurons has increasingly gained attention during the last years (Clemens et al., 2011; Brill et al., 2013; Campbell et al., 2013; Guo and Ritzmann, 2013; Saha et al., 2013; Duer et al., 2015).

The crab *Neohelice granulata* has been extensively used as a model animal in different fields of biology, from ecology to neurobiology (Spivak, 2010). It is a highly visual semiterrestrial crab that inhabits densely populated mudflat environments. In nature, the crab is regularly engaged in social interactions that include burrow defense, courtship, chasing after smaller individuals and being chased by larger ones (Fathala and Maldonado, 2011; Sal Moyano et al., 2016; Tomsic et al., 2017; Gancedo et al., 2020), all activities that are guided by sight. The crab also uses vision to detect and avoid aerial predators (Magani et al., 2016). Accordingly, vision plays a leading role in the behavior of this animal.

Neurobiological studies on *Neohelice* mainly focused on the crab’s escape response to visual threats and encompassed different aspects such as visuomotor transformation, response modulation, and learning and memory. The studies were performed with a variety of methodologies that include behavioral analyses, neuroanatomy, pharmacology, molecular biology, electrophysiology, and calcium imaging (for reviews see Tomsic and Romano, 2013; Tomsic, 2016; Tomsic et al., 2017). An important step in the establishment of the crab as an invertebrate model for studying the neural control of behavior has been the identification and characterization of a group of giant neurons from the lobula (3rd optic neuropil of arthropods), which were shown to be key elements for visually-elicited avoidance behaviors. The achievements had been possible due to the unique experimental advantages offered by this crab to perform stable intracellular recordings from brain neurons in the practically intact and awake animal (e.g., Berón de Astrada and Tomsic, 2002; Scarano et al., 2018). Four different classes of lobula giant (LG) neurons had been studied. The different classes exhibit commonalities and differences. Morphologically, they all have wide dendritic trees that extend across tangential layers of the lobula, from where they collect visual information provided by the columnar elements of the retinotopic mosaic (Sztarker et al., 2005; Berón de Astrada et al., 2013) and their axons project through the protocerebral track toward the midbrain (Berón de Astrada and Tomsic, 2002; Medan et al., 2007). A common physiological signature to all LG neurons is their response plasticity on repeated motion stimulation. Such plasticity has been shown to underlie part of the short- and long-term memory traces induced by visual training (Tomsic et al., 2003; Sztarker and Tomsic, 2011). LG neurons also share the ability to integrate binocular information (Sztarker and Tomsic, 2004; Scarano et al., 2018) and three classes integrate visual with mechanosensory information from the animal’s legs (Berón de Astrada and Tomsic, 2002; Medan et al., 2007). Beyond these commonalities, the four LG classes show substantial differences. Two classes have dendritic trees extended across a single tangential layer of the lobula and, therefore, had been named Monostratified Lobula Giants type 1 and type 2 (MLG1 and MLG2, respectively), whereas the other two classes have dendritic trees extended over two tangential layers and, hence, were named Bistratified Lobula Giants type 1 and type 2 (BLG1 and BLG2). MLG1s form an ensemble of 16 elements distributed across the lateromedial axis of the lobula, mapping the 360° of azimuthal space. These elements are thought to convey information about objects position and object motion dynamics in terms of population code and activity code, respectively (Oliva and Tomsic, 2014; Medan et al., 2015). Contrasting, MLG2 is likely a unique element, with a receptive field covering the entire visual space (Medan et al., 2007). This neuron has been shown to play a central role in regulating the animal’s speed of run according to the visual dynamic of approaching stimuli (Oliva and Tomsic, 2016). The BLG1 class is composed of a discrete number of elements (Medan et al., 2007; Scarano et al., 2018), which might participate in encoding information regarding stimulus elevation (Tomsic, 2016). The BLG2 is a very large neuron, likely a single element, with an extensive receptive visual field (Medan et al., 2007). Contrasting with the three previous classes, which responses to looming stimuli consist of a firing rate increase that follows the dynamic of image expansion, the BLG2 neuron strongly responds at the very beginning of looming stimulation and inactivates with further image expansions. The time course of the BLG2 activity to looming stimuli approximately coincides with transient freezing observed in the animal before initiating the escape (Oliva, personal communication), suggesting a role of this neuron in that behavioral component (Tomsic et al., 2017).

Considering their complex morphology, multisensory integration, plasticity properties, and the correspondence observed between their activity and the behavioral responses under different circumstances, the group of the LG neurons is thought to operate as a decision-making node for several important aspects of the visually-guided avoidance behavior. Yet, the connectivity among the different LG neurons is still unknown. Here, we began to bridge this gap by performing multielectrode recordings of neurons from the lobula neuropil of the crab.

## Materials and Methods

### Animals

The animals were adult male *Neohelice granulata* crabs 2.7–3.0 cm across the carapace, weighing approximately 17 g, collected in the rías (narrow coastal inlets) of San Clemente del Tuyú, Argentina. The crabs were maintained individually in glass jars filled to 2 cm depth with artificial seawater prepared using hw-Marinex (Winex, Hamburg, Germany), salinity 10–14%, at a pH of 7.4–7.6 and maintained within a range of 22–24°C. The holding and experimental rooms were kept on a 12 h light/dark cycle (lights on 7:00 AM to 7:00 PM) and the experiments were run between 8:00 AM and 7:00 PM, two to seven days after the animals’ arrival to the laboratory.

### Visual Stimuli

This study represents our first approach to using multielectrode recording in the crab. For this reason, we included in our experiments stimuli that proved to be effective both for identifying different lobula neurons (Medan et al., 2015; Tomsic et al., 2017; Scarano et al., 2018, 2020) and for eliciting a variety of behavioral responses in this animal, such as escape response (Oliva and Tomsic, 2012; Scarano and Tomsic, 2014), predatory response (Gancedo et al., 2020) and optomotor response (Barnatan et al., 2019). Computer-generated visual stimuli were projected on a computer screen (Samsung S20C300L) placed at a distance of 20 cm, covering the frontolateral right side of the animal. The screen was housed inside a Faraday cage with opaque covers to prevent outside visual stimuli from reaching the animal. Visual stimuli were of three different types: (a) black squares of three different sizes that moved at three different heights; (b) a grating pattern; and (c) a looming stimulus. The first two types moved rightward or leftward over a white background, covering a translation distance of 37 cm (spanning a visual arc of 85° from the crab point of view), at a speed of 18 cm/s (corresponding to a retinal speed at the center of the screen of near 52°/s). According to their size, square stimuli were named small, medium, and large (1.5 × 1.5 cm, 3 × 3 cm, and 6 × 6 cm, subtending angles at the center of the screen of approximately 6, 12, and 17 square degrees respectively). These stimuli moved at the level of the horizon and 17° above and below the horizon. The grating consisted of a pattern of black and white vertical bars of 6 × 24.5 cm (the retinal subtended angle at the center of the screen 17 × 63°) extended over the whole screen. The remaining stimulus was a looming stimulus, a 2D representation of a black square object approaching the crab at constant velocity with an l/v ratio of 120 ms (expanding from 4° to 60° in 3.36 s). Stimulation consisted of four consecutive rounds of stimuli presentations, each round encompassing the 21 different stimuli (including size and direction variations) delivered in random order. The time between stimuli presentations was no less than 45 s. Visual stimuli were generated using Matlab custom-built software. To assess the timing of the stimuli on the neuronal recordings we used an Arduino, which sent a TTL pulse at the start and the end of the stimulus to the electrophysiological interface board.

### Animal Preparation

**Figure 1:**
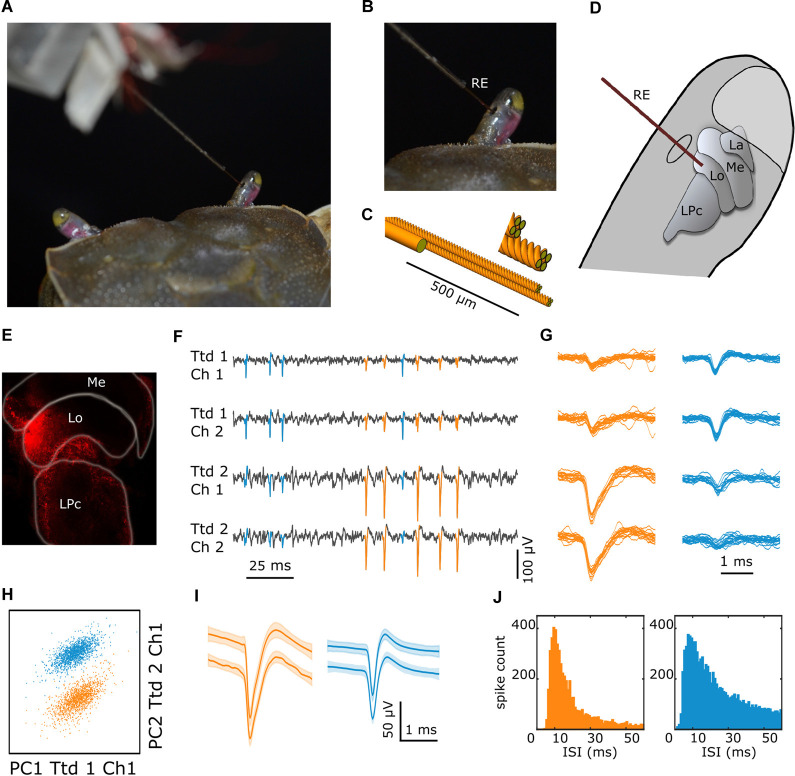
Multielectrode recording procedure and data processing. **(A)** The dorsal part of a crab as viewed from behind with the electrode entering the right eyestalk from its medial side. **(B)** Closer view of the eyestalk with the recording electrode (RE) passing through a small hole cut in the cuticle. **(C)** Detail of the custom-made 8-channel multielectrode, consisting of a pair of tetrodes (four twisted 12 μm tungsten wires each) with their tips separated 50–100 μm and the thicker (50 μm) reference wire 500 μm from the tip. **(D)** Scheme of the eyestalk with the retinotopic neuropils (La: lamina, Me: medulla, Lo: lobula), the lateral protocerebrum (LPc), and the multielectrode targeting the lobula. **(E)** Confocal image showing the spot in the lobula left by the dye at the tip of the electrode. **(F)** Fragment of a recording showing electrical signals obtained in four different channels, with the two upper and two lower traces corresponding to different tetrodes. Signals highlighted in blue and orange identify spikes of two different units. **(G)** Superimposed spikes of each unit were obtained in the four channels. **(H)** Scatter plot of the first two principal components analyses of the waveforms from tetrodes one and two, where cluster membership is indicated by color. **(I)** Mean ± SEM waveforms of the two units from the spikes recorded during 10 min preceding and following the experiment (upper and lower traces, respectively). **(J)** Interspike interval histograms for all spikes of the two sorted units.

The crab was firmly held in an adjustable clamp which allowed free movements of the walking legs but reduced movements of the chelae (Berón de Astrada and Tomsic, 2002). The eyestalks were cemented to the carapace at an angle of approximately 50° from the horizontal line, which corresponds to their normal seeing position (Scarano et al., 2018). A small hole on the medial side of the eyestalk cuticle was drilled to introduce the electrode at the level of the lobula (Figures 1A–D). After this, the clamp with the crab was mounted inside the recording setup using magnetic holding devices. The multielectrode was then positioned and advanced through the opening in the cuticle. All the recordings were taken from the right eyestalk.

### Multielectrode Construction and Recording Devices

We used a custom-made eight-channel multielectrode. It consisted of a pair of tetrodes (four twisted 12 μm tungsten wires each) and a reference (single 50 μm tungsten wire). First, both tetrodes and the reference were slid into a metal capillary which was fixed on a small plexiglass plate controlled by a micromanipulator. Then, the tetrode tips were cut at a 45-degree angle with carbide scissors to improve tissue penetration and the two bundles were glued together using methacrylate, with their tips separated by 50–100 μm. The reference was also glued to the tetrodes approximately 500 μm from their tips, helping to straighten the ensemble (Figure 1C). Each electrode impedance at 1 kHz was adjusted to approximately 150 KΩ using gold electroplating. The plexiglass plate contained the plugs for connecting every independent wire, which in turn were connected to the amplifier (Intan RHD2132 16-channel amplifier board). An interface board (RHD2000 USB interface board) allowed to simultaneously acquire neuronal data and the timing of visual stimuli (TTL pulses indicated the start and end of each visual stimulus). Data were acquired at 30 kHz and recorded on a PC using Intan software (RHD2000 Evaluation System Software).

### Experimental Protocol

Once the multielectrode was inside the eyestalk, it was gently moved forward until clear spike signals to noise ratio were obtained. Then, a rapid preliminary test was performed by presenting a moving stimulus to detect evident neural responses. If satisfactory responses were not observed, the electrode was advanced until a clear-cut response to motion stimulation was achieved. After some practice, we were able to get suitable responses quite easily. However, at this stage, observable responses usually contained the activity of different neuronal units, which could only be separated and distinguished after processing the data off-line. Once the electrode was in a position from which we decided to perform the experiment, we put down the curtain at the front of the Faraday cage and waited for 10 min to start recording. Our experimental protocol included continuous recording during the full sequence of visual motion stimuli presented four times (as described above), plus 10 min of basal activity at both ends of the recording session. These periods of basal activity were used to assure that the signals remained the same across the entire recording.

To confirm that the multielectrode was actually targeting the lobula, in a few experiments we dipped the tips of the electrodes into a concentrated solution of Dil (1,1′-dioctadecyl-3,3,3′,3′-tetramethylindocarbocyanine perchlorate) before approaching the tissue. After the recordings, we dissected and prepared the optic ganglia to be observed with the confocal microscope.

### Data Processing

There are three main steps involved in spike sorting: spike detection, feature extraction, and spike clustering based on combinations of extracted features (Takekawa et al., 2010). Data were first high pass filtered (a median-based filter with a window half-length of 90 samples), then the spikes (Figure 1E) were detected using a voltage threshold of 6 SD, and finally, a principal component analysis (PCA) was performed to each waveform. The waveform time window was set from −0.8 ms before to 1.2 ms after either positive or negative peak amplitude (Figure 1F). All these steps were done using NDManager (Hazan et al., 2006). Automated clustering was performed with the program KlustaKwik (version 1.5^1^) and then imported into Klusters (Hazan et al., 2006) for further classification and refinement.

Because spike sorting is sensitive to misclassification (Harris et al., 2000; Joshua et al., 2007; Quiroga et al., 2007), we considered a series of visual tests on the output of automated spike sorting routines that address whether a single cluster of waveforms is self-consistent with a single neuron (Hill et al., 2011). These were as follows.

(a) Inspect the waveforms: for every sorted unit, the spike shapes were superimposed to make false-positive sorting visible. We cleaned false data manually.
(b) Inspect for stationary: for each unit, if the spontaneous activity shifted noticeably during the experiment, data after the shift were excluded from the analysis. We also checked that the mean waveform obtained during a baseline period in the beginning and the end of the experiment was unchanged.
(c) Distribution of interspike intervals (ISIs): very short ISIs (<1–2 ms) are unlikely to occur in a single unit because of its refractory period, so an ISI histogram with a substantial number of occurrences at small ISIs suggests that multiple neurons may be included within a cluster.

After completion of these analyses, clusters were considered to represent spikes of individual neurons.

### Analysis of Responses to Visual Stimuli and Classification of Units

Following the identification of the spikes corresponding to individual neurons, the responses to visual stimuli presentations were analyzed. Peri event time histograms (PETH) were computed for each neuron with a bin size of 10 ms. To calculate the instantaneous firing rate, every single raster built on the spike times was smoothed using a Gaussian kernel with a width of 100 ms (10 bins). PETH was constructed with four trials for each stimulus type. In this way, we obtained the response profiles of each unit for all the stimuli.

### Further Analyses

In addition to the response profile to visual stimulation, several features of the extracellularly recorded units were examined. These included spontaneous firing rate, bursting pattern, spike duration, spike asymmetry, the amplitude ratio of the negative and positive peaks and recovery time, as well as features of the auto-correlogram such as the time from peak to peak. Possible interactions between simultaneously recorded units were analyzed using cross-correlograms. Excitatory connections were associated with short-latency and -duration sharp peaks in the cross-correlogram, while short-latency troughs were considered to be due to inhibition (Csicsvari et al., 1998).

## Results

### General Description

We recorded the neural activity of 93 units from the lobula in 27 animals. The number of reliably identified units per experiment varied between 1 and 8. Figure 1 shows the method of recording and the general procedure of spike sorting and clustering illustrated on data from a particular experiment. Figure 1F shows a recording example where the spike activity of two different units can be observed. The two upper and lower traces correspond to channels of different tetrodes. Note that the blue marked spikes are larger in the two upper channels than in the lower ones, whereas for the orange marked spikes the relation is inverted. This becomes clearer when the waveforms of several individual spikes are superimposed (Figure 1G). PCA performed over the entire recording time across all eight channels allowed to distinguish two signal clusters in this particular recording (Figure 1H). This result was supported by a series of visual tests that we applied to further address whether a single cluster of waveforms is self-consistent with a single neuron (see “Materials and Methods” section). Figure 1I shows the mean ± SEM waveforms of spikes obtained during the first and last 10 min of the experiment (upper and lower traces, respectively) for both units. Despite the samples were taken more than one hour apart, the waveform of each neuron remained unchanged. Also, the distribution of interspike intervals (ISIs) depicted in Figure 1J confirms that none of the units reflect refractory period violations (i.e., ISI < 1–2 ms).

### Distinct Units Exhibit Differential Response Preferences for Visual Stimuli

Once the spikes of distinct units had been sorted and clustered, we analyzed the responses of each unit to the presentation of the visual stimuli. A first analysis, based on the ratio between the firing rates measured over a 2 s time window immediately before and after the initiation of motion stimulation for all the stimuli, showed that 86% of the recorded units responded with an increase of their firing rate, 4% with a reduction and 10% showed no change. An equivalent result was observed when the different stimuli were analyzed separately.

**Figure 2:**
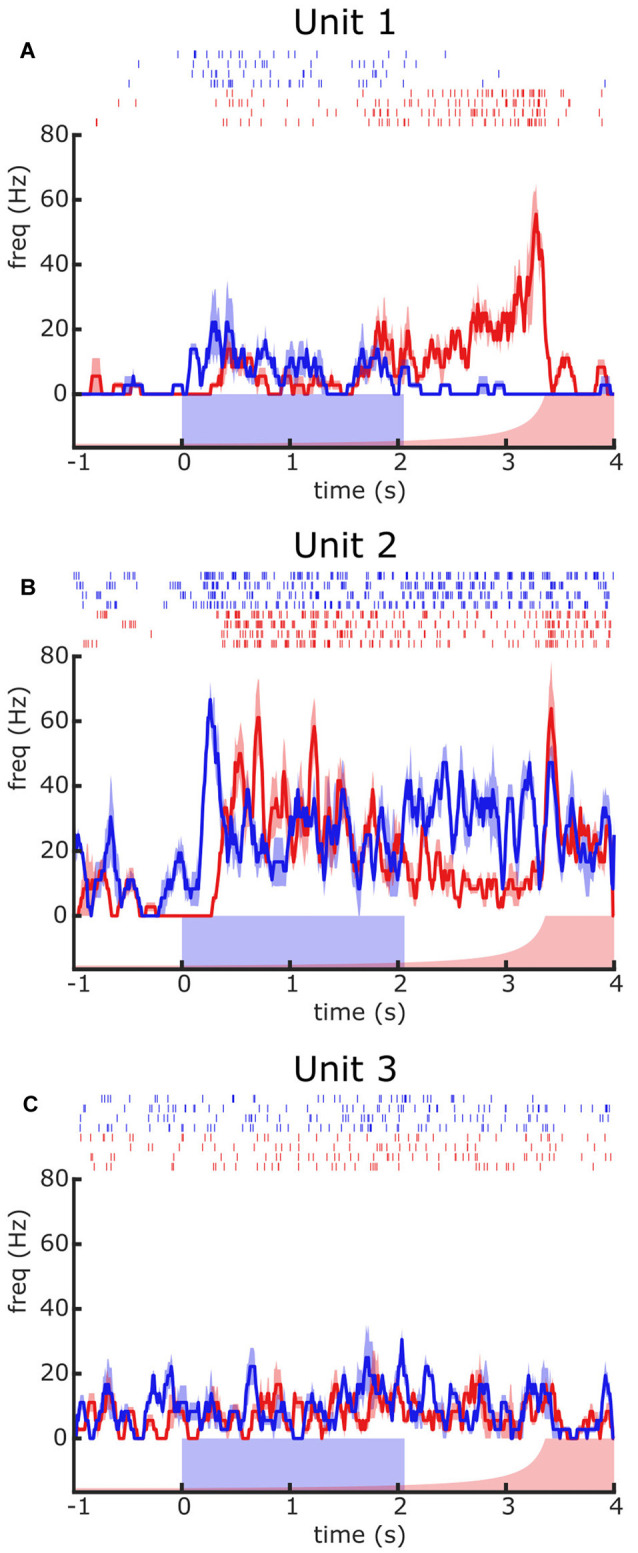
Contrasting responses of different units to visual presentations of a looming (red) or a moving square (blue) stimulus. **(A–C)** Responses of three different units recorded from different crabs. For each unit, the raster plot depicts the temporal course of elicited spikes (response) to four presentations of each stimulus type. The traces correspond to the mean ± SEM firing frequency. The blue rectangle over the X-axis represents the motion duration of the square stimulus. The red curve represents the angular size of the looming stimulus, which remained stationary until time zero when it started to expand. Further details are in the text.

Responses to square stimuli of different sizes and elevations differed in intensity (being the most effective the large square moving at the level of the horizon), but not in their profiles. Therefore, our description concentrates on the size and elevation that elicited the strongest response. Figure 2 illustrates the responses of three different units to the large square stimulus (blue) and the looming stimulus (red). Raster plots reflect the responses recorded across four trials and the traces are the mean ± SEM. Each unit exhibits a different response profile. Unit 1 shows a moderate increase of firing rate to the moving square that ends when the stimulus stops moving, whilst it shows a progressive increase of firing rate to the looming stimulus that nearly matches the image growing and suddenly suppresses with the end of the expansion. Unit 2 shows a response to the square lead by a prominent peak of firing rate followed by a steady-state that extends beyond the stimulus end, whereas the response to looming consists of an early substantial increase of firing that progressively decays with the image expansion and is followed by a marked rebound at the end of the expansion. Unit 3 shows no appreciable response to either the square or the looming stimulus. These results demonstrate the feasibility of disclosing and classifying units based on their response preference for distinct visual stimuli, an issue that we further elaborate throughout the next sections.

### Identification of LG Neurons

**Figure 3:**
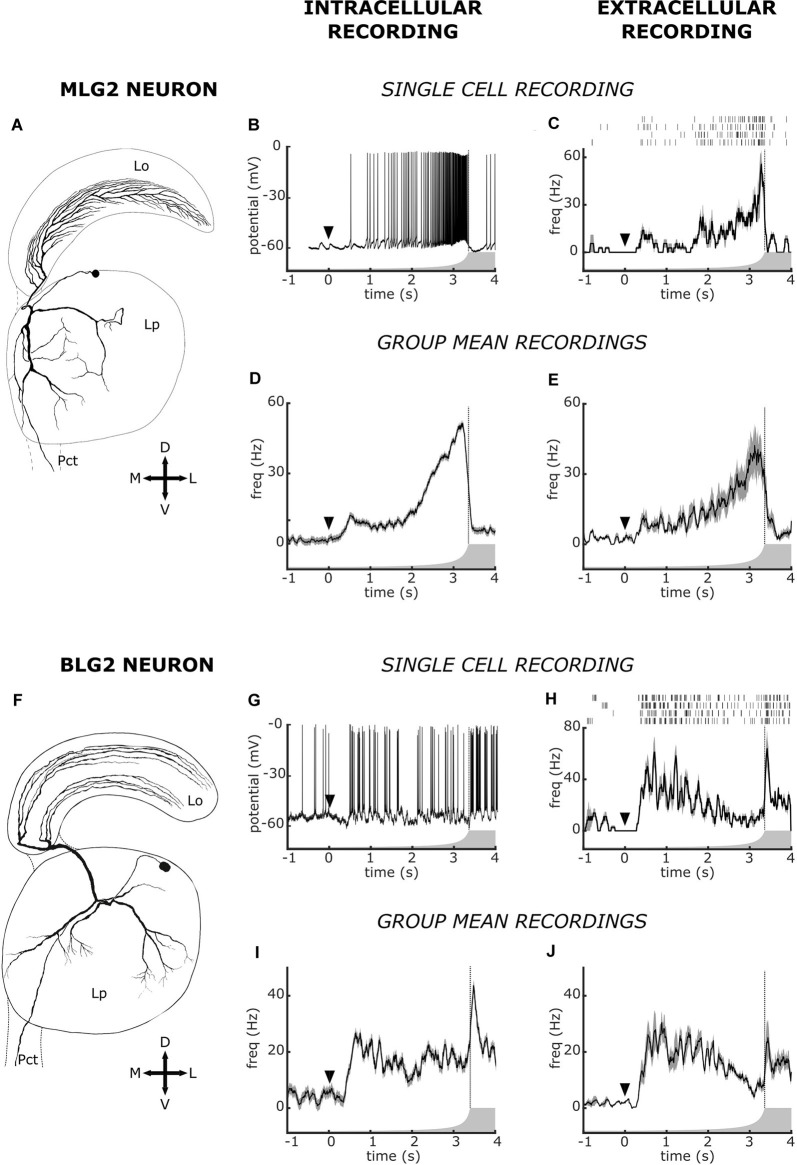
Intracellular and extracellular recorded responses to a looming stimulus of Monostratified Lobula Giant type 2 neuron (MLG2) and of Bistratified Lobula Giant 2 neuron (BLG2). **(A–E)** Data on MLG2. **(F–J)** Data on BLG2. **(A,F)** Morphology of the two neuronal types. **(B,G)** Examples of intracellular recorded responses. **(C,H)** Examples of extracellular recorded responses. For each unit, the raster plot shows responses to four presentations of the looming stimulus and the traces are the mean ± SEM. **(D,I)** Mean ± SEM of intracellularly recorded responses (as those shown in panels **B,G**) obtained from different animals. In panels (**D,I**) the number of averaged animals (one mean response per animal) is 37 and 13, respectively. **(E,J)** Mean ± SEM of extracellularly recorded responses (as those shown in panels **C,H**) obtained from different animals. In panels (**E,J**) the number of animals (one mean response per animal) is five and eight, respectively. The arrowhead at time zero marks the beginning of stimulus expansion, which is represented by the curved profile of the gray form. The vertical dotted line denotes the end of the expansion. The cell morphologies shown in panels (**A,F**) are from Medan et al. (2007). Data shown in panels (**B,D**) have been modified from Oliva and Tomsic (2016). Data in panels (**G,I**) have been modified from Oliva (2010).

**Figure 4:**
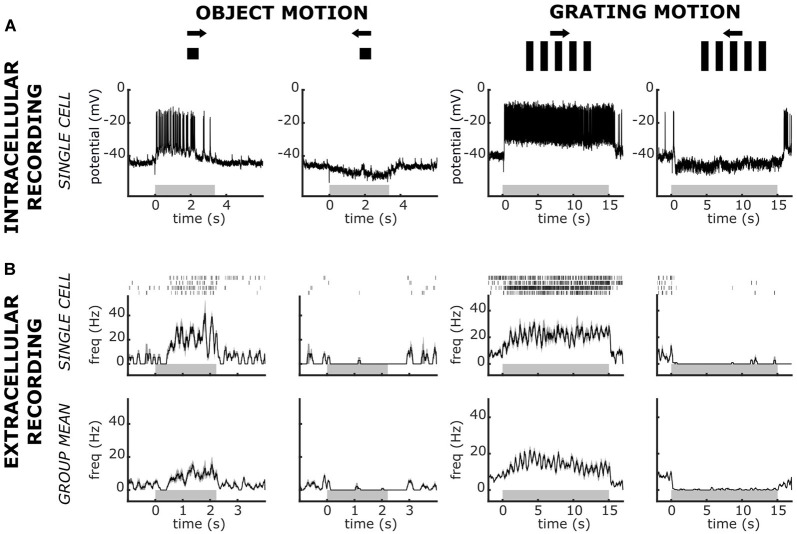
Intracellular and extracellular recorded responses of Lobula Complex Directional Cells (LCDC) to visual presentations of a single moving object or a grating pattern. Responses to the rightward and leftward motion were recorded for both stimulus types. **(A)** Responses from a single intracellularly recorded neuron. **(B)** Responses from extracellular recorded units. Upper panels: responses of a single unit. Raster plots show responses to four presentations of the stimulus and the traces are the mean ± SEM. Lower panels: Mean ± SEM obtained from five animals (one mean response per animal). Gray horizontal rectangles stand for the time of stimulus motion. Note the differences in the scale times among panels. Data in panel **(A)** have been modified from Scarano et al. (2020). See the text for further details.

The multielectrode extracellular technique prevents the morphological identification of the recorded neurons. Consequently, in most studies, whether they are carried out in vertebrate or in invertebrate animals, the identity of recorded neurons is essentially unknown. The best approximation for neuronal identification resides on previous knowledge of neurons housed in the area from where the extracellular recording is taken, namely in the possibility of establishing correspondences between patterns of activity recorded extracellularly with those seen in neurons that had been characterized intracellularly. Yet, even in invertebrates, the strategy of identifying neurons by comparing extracellular data with intracellular data proved not to be easy (Bhavsar et al., 2015). We were confident that this could be achieved in the crab because the lobula is an easily targeting neuropil that contains several classes of morphologically identified and physiologically characterized neurons of exceptionally large size, the LG neurons. Results shown in Figures 3, 4 substantiate our assumption. Figure 3 allows comparing the response profile to a looming stimulus obtained by intracellular recording from neurons MLG2 and BLG2 with similar responses from units obtained by extracellular recording. The characterizations performed by intracellular recording followed by cell staining have shown that these two neurons arborize across the whole lobula as well as in several regions of the lateral protocerebrum (Figures 3A,F), and their physiological receptive fields cover the entire visual field of the animal (Medan et al., 2007). However, the response of these two cells to looming stimuli was very different. On one hand, the MLG2 neuron increases the firing rate according to the dynamic of image expansion (Oliva et al., 2007; Oliva and Tomsic, 2016). This is illustrated in the intracellularly recorded trace of Figure 3B. A remarkably similar profile of spiking activity was found in some extracellular recorded units, as the one shown in Figure 3C. The correspondence between the activity of intracellularly and extracellularly recorded elements becomes more evident and reliable when the mean response from several units with similar responses recorded from different animals are compared (intracellular *n* = 37, extracellular *n* = 5, Figures 3D,E, respectively). The BLG2 neuron, on the other hand, has been shown to respond to looming stimuli with an early substantial increase of firing rate, followed by a steady-state or even a gradual suppression during the rapid phase of stimulus expansion and a transient rebound of high-frequency firing just after the end of the expansion. This can be observed in the intracellular recorded response of Figure 3G. Again, we found a similar response profile in some of the extracellular recorded units, like the one shown in Figure 3H. The resemblance between the response of intracellularly recorded BLG2 neurons and the response of some extracellularly recorded units can be appreciated by comparing the mean response profiles of neurons obtained from different individuals (intracellular *n* = 13, extracellular *n* = 8, Figures 3I,J, respectively).

In addition to the response profiles to visual stimulation just described, our knowledge on the activity of LG neurons acquired by intracellular recordings allows comparing other features of the extracellularly recorded units. For example, the MLG2 has been shown to exhibit a spontaneous activity made of individual spikes, whereas the BLG2 was shown to display spontaneous activity characterized by bursts of spikes (tables 1 and 2 in Medan et al., 2007). In the raster plot of Figure 3C, the spontaneous activity preceding the start of the looming contains isolated spikes. On the other hand, the raster plot of Figure 3H shows a spontaneous activity made of bursts. We then analyzed the firing pattern of all our MLG2 and BLG2 classified units, by calculating the percentage of total spikes that occurred as bursts of three or more spikes with an interspike interval of less than 15 ms (Longden et al., 2017). The mean ± SEM percentage for the MLG2 units (*n* = 5) was 3.3 ± 1, 4 and for the BLG2 units (*n* = 8) was 15.7 ± 2.7, a difference that was statistically significant (*p* < 0.01, Student’s *t*-test). Therefore the pattern of bursting activity provides further confidence for our classification of these extracellularly recorded units as MLG2 and BLG2 neurons.

### Identification of Directional Sensitive Neurons

The four LG classes of neurons have scarce or null motion directional preferences (Medan et al., 2007). Recently, we have described a novel group of large neurons of the crab that exhibit a remarkable directional preference for visual stimuli moving along the horizontal plane. Because of their arborizations in the lobula and the lobula plate, we called these cells lobula complex directional cells (LCDC; Scarano et al., 2020). The LCDC response to a moving square is characterized by a clear increase of spike discharge in one direction, the so-called preferred direction, and a hyperpolarization with suppression of spontaneous spikes in the opposite direction called the null direction. Some LCDC neurons have been shown to present sustained responses to optic flow in the preferred as well as in the null direction (Scarano et al., 2020). Figure 4A shows the intracellularly recorded response of an LCDC to a single object and a grating pattern moved in opposite directions. In our extracellular recordings, we have identified units that responded as LCDC neurons. The performance of one such unit is illustrated in Figure 4B (upper row), where the responses to the four trials (raster plot) recorded for each stimulus condition and the averaged response from these trials (trace) are depicted. Figure 4B (lower row) shows the mean responses from five recorded units, which responses were similar to those of the unit depicted in the upper panels. The results show the remarkable directional sensitivity of these units, consisting of a sustained increase in the firing rate to one motion direction as well as a sustained suppression of the spontaneous spike activity in the opposite direction. The equivalence between these response profiles and those obtained with intracellular recordings from LCDC neurons strengthens our initial confidence in the feasibility to recognize in extracellular recordings some of the previously identified elements of the crab’s lobula neuropil.

### Additional Commonalities Within Groups of Identified Units

**Figure 5:**
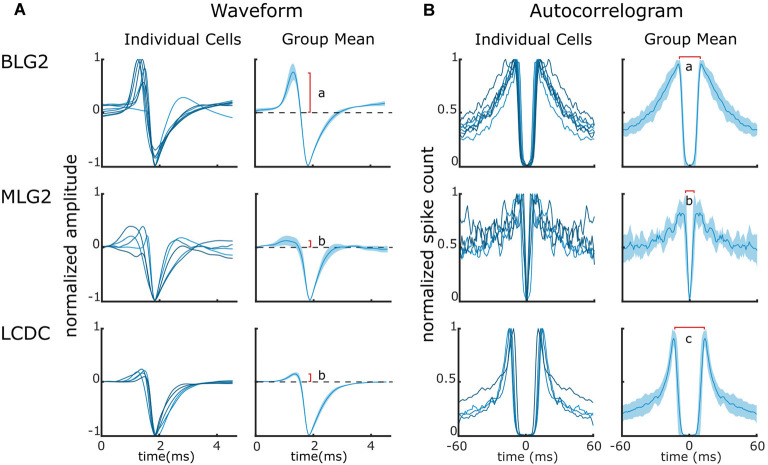
Waveforms and auto-correlograms of BLG2, MLG2, and LCDC neurons. **(A)** Waveforms of individual units (left panels) and Mean ± SEM waveforms (right panels) of the three identified neuronal classes. The red vertical line indicates the mean size of the waveform positive phase. Different letters denote a significant difference. **(B)** Auto-correlograms of individual units (left panels) and Mean ± SEM (right panels). The red horizontal line indicates the mean time between the peeks of the spike count. Different letters denote a significant difference. Further details are in the text.

The identification of extracellularly recorded units as MLG2, BLG2, or LCDC neurons just described was based on the recognition of particular patterns of activity in response to presentations of specific visual stimuli. To further investigate the reliability of this criterion for picking out elements of a particular neuronal class, we analyzed the consistency of the waveforms and the auto-correlograms among the units of each particular group (Figures 5A,B, respectively). Contrasting with the analysis of the response profiles during the presentation of visual stimuli, which comprised just a small fraction (4%) of the entire recording time, the mean waveform and the auto-correlogram take into consideration all the spikes sorted for each unit during the whole duration of the recording. Figure 5A (left column) shows the mean waveform of each unit, for all those units that were identified by their responses as MLG2, BLG2, or LCDC. A cursory inspection allows seeing that within each group the waveforms have rather similar shapes (with one exception in the BLG2 group) and that the shapes differ among the groups. In particular, BLG2 units exhibit a conspicuous positive phase that is barely observable in MLG2 and LCDC units. The differences become more evident when the mean waveforms of the groups (obtained from the individual means) are compared (Figure 5A, right column). A comparison of simple features of the mean waveforms, such as the relative magnitude of the positive phase (red vertical line), reveals statistical differences between groups tagged with different letters (BLG2 vs. MLG2, *p* < 0.01; BLG2 vs. LCDC, *p* < 0.01; MLG2 vs. LCDC, *p* > 0.5, one way ANOVA followed by Tukey’s test).

Figure 5B (left column) shows the auto-correlograms of all those units classified as MLG2, BLG2, or LCDC. The auto-correlograms of units within each group are qualitatively more similar between them than to those of units from the other groups. Figure 5B (right column) shows the mean ± SEM of the auto-correlograms of the three cell classes. A simple comparison of the time that separates the peeks of higher probability (red horizontal line) shows statistical differences among all groups (BLG2 vs. MLG2, *p* < 0.01; BLG2 vs. LCDC, *p* < 0.05; MLG2 vs. LCDC, *p* < 0.01, one way ANOVA followed by Tukey’s test).

The coherence found in the waveforms and in the auto-correlograms among the units that had been classified by their responses to visual stimulation as belonging to a specific class provides strong support to the use of those responses as a solid criterion for recognizing specific neuronal classes within the lobula of the crab. Moreover, the results show that a proper recognition of a unit as an MLG2, BLG2 or LCDC, should satisfy the three criteria identified here, namely: (i) a particular response profile to visual stimuli; (ii) a predictable waveform shape; and (iii) an expected outline in the auto-correlogram.

### Functional Connections Between Units

The simultaneous recording of activity from different units performed with multi electrodes offers the possibility of disclosing functional relationships between them. These interactions are typically visualized through cross-correlation analyses (Barthó et al., 2004). We analyzed cross-correlograms built with the spikes recorded during the whole duration of the experiment, thus comprising periods of spontaneous activity as well as of evoked activity (stimuli presentations). We also examined the cross-correlograms built exclusively with the spikes generated during the stimulation periods but, because the sum of these periods represents only about 4% of the entire recording time, the numbers of events were insufficient for the analyses. Cross-correlograms (and auto-correlograms) built on the total recorded spikes and those built on the periods of spontaneous activity looked very similar.

**Figure 6:**
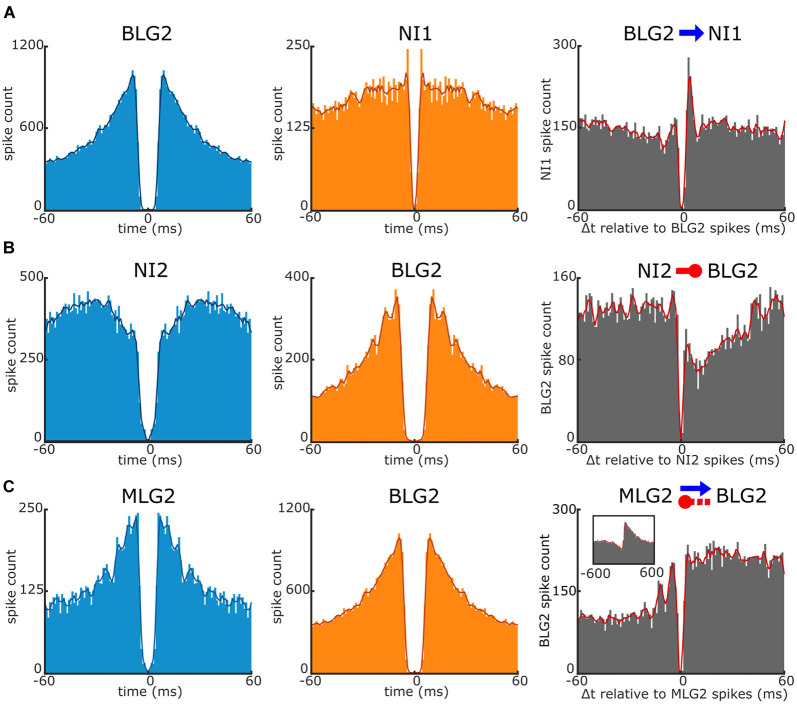
Interaction between a pair of units recorded simultaneously. Examples of interactions revealed by cross-correlogram analyses of three pairs of units. The left and middle panels are the auto-correlograms of the units corresponding to the cross-correlograms shown on the right panels. **(A)** Short-latency monosynaptic excitatory interaction of the BLG2 cell on a non-identified unit 1 (NI1; blue arrow). Note the large, sharp peak at near 4 ms in the cross-correlogram. The reference event (*time 0*) is the spike of the BLG2. **(B)** Short-latency monosynaptic inhibitory interaction of a non-identified unit 2 (NI2) on the BLG2 neuron (red circle-ended line). Note the strong and immediate suppression of target spikes. The reference event is the spike of the NI2 neuron. **(C)** Complex reciprocal interactions between the MLG2 and BLG2 neurons (blue arrow and red circle-ended line). Reference event: the spike of the MLG2 unit. In the inset, data are shown over an extending period. Note the long-lasting excitation of the BLG2 and the delayed suppression of the MLG2 spikes. Further explanations are in the text.

Of the 155 cell pairs analyzed in our experiments, we found that near 15% showed apparent interactions. Figure 6 illustrates three different types of interactions found in our recordings. For the cross-correlograms (gray panels) the reference event (time 0) is the spike of the corresponding unit which auto-correlogram is shown in light blue. Figure 6A shows the auto-correlograms of a BLG2 cell and a non-identified cell 1 with the corresponding cross-correlogram. The cross-correlogram contains a clear and narrow short-latency peak (<5 ms), indicating that the BLG2 presynaptic neuron was an excitatory cell. Figure 6B shows the auto-correlograms of a non-identified cell 2 and a BLG2 cell. The cross-correlogram of these cells exhibits a short-latency suppression (<10 ms), indicating that the non-identified presynaptic neuron was inhibitory on the BLG2 neuron. Finally, Figure 6C presents the auto-correlograms corresponding to an MLG2 and a BLG2. In this case, the cross-correlogram shows both a short-latency sustained peak and a delayed trough, suggesting that the elements of the pair were mutually connected. The MLG2 was excitatory on the BLG2, whereas the BLG2 exerted an indirect (delayed) inhibition on the MLG2. The short-latency and long-lasting peak and the delayed inhibition can be better appreciated on the extended timescale shown in the figure inset.

The inhibitory effect of the BLG2 on the MLG2 is in agreement with what is observed in the response profiles to the looming stimulus of these neurons (Figure 3), i.e., the time of higher firing frequency of the BLG2 at the beginning and the end of the stimulus expansion coincides with the time of lower spike frequency of the MLG2, which finds its maximal rate when the BLG2 has its trough. Following similar reasoning, it would be expected that the cross-correlogram had revealed an inhibitory effect of MLG2 on the BLG2, but this was not the case. Far more experiments are needed to unravel the complex functional connections existing in the microcircuit formed by the large neurons of the lobula.

## Discussion

The crab *Neohelice* is a well established invertebrate model for investigating the neurobiology of visually guided behaviors, including learning and memory processes. Over the last two decades a great deal of knowledge about different lobula giant neurons that play central roles in the crab’s escape behavior from visual threats has been acquired (Tomsic et al., 2017). The characterization of these LG neurons has been made by *in vivo* intracellular recording and staining. The present account describes results obtained by multielectrode extracellular recording for the first time. This study aimed to seek out the possibility of identifying LG neurons from the extracellular recorded units based on the similarity of responses to those recorded intracellularly with a variety of visual stimuli. The results show that the expectation was fulfilled. Moreover, by simultaneously recording from multiples units we proved the feasibility of disclosing the interactions between them.

### Characteristics of the Multielectrode Extracellular Recording in the Crab

Extracellular recording is the oldest and most common method for recording electrical activity across populations of neurons in awake behaving animals, from invertebrates to human primates. Yet simple criteria for acceptable data, particularly concerning claims of single-unit responses, are largely missing. Such criteria are critical since interpretations of spike trains that are based on inadequately sorted units can lead to erroneous claims on neural coding (Hill et al., 2011). Because this is our first study using this methodology, we adopted conservative spike sorting criteria (see “Materials and Methods” section). This reduced the number of potentially analyzable units per experiment but increased our confidence by relying on units whose signals were most conspicuous. On average, we have considered for analyses 3.4 units per experiment (range 1–8, median 4), which is comparable to the average of 2.7 units (56 units from 21 preparations) recorded from the central complex of the cockroach using a similar pair of 12 μm wire-bundle tetrodes (Guo and Ritzmann, 2013).

Crabs offer the singular advantage of allowing to perform stable intracellular recording in the practically intact animal, which following the experiment remains perfectly healthy (e.g., Tomsic et al., 2003). This holds for multielectrode recording. The stability of these recordings is illustrated in Figure 1I, which shows that the unit’s waveforms remained unchanged throughout the experiment, even though the electrode was not affixed to the carapace of the crab and that the animal sporadically moved its legs. The recording stability obtained under such conditions seems to warranty the feasibility of recordings from the freely moving animal. The chances for this are also supported by the fact that after the recording all the animals used in this study remained healthy.

### Extracellular Recognition of Previously Identified Neurons

Intracellular recording and staining allow to unequivocally establish fundamental aspects of the cell physiology together with the cell’s exact location and morphology. Therefore, the possibility of associating extracellularly recorded units with intracellularly well-characterized elements is of paramount importance. Yet, extracellular multichannel recordings in arthropods have mostly been made from unidentified cells, and attempts to recognize specific neurons based on matching the responses to particular stimuli with those previously obtained from intracellular recordings have largely failed (e.g., Bhavsar et al., 2015). By recording with the duo-tetrode from the lobula we were able to confidently identify two of the four types of LG neurons that have been described so far (e.g., Medan et al., 2007), as well as the recently described directional giant neurons (Scarano et al., 2020). Among the four classes of LG neurons, the MLG2 and BLG2 recognized in the present study are the largest lobula neurons, whose arborizations profusely extend all over the neuropil (Medan et al., 2007). Similarly, the so-called lobula complex directional cells (LCDC) present extensive arborizations within the lobula (Scarano et al., 2020). These characteristics most certainly facilitated recording from these elements. The classification of recorded units as MLG2, BLG2, or LCDC was based on the similarity between the patterns of responses (temporal course and intensity of firing frequency) obtained extracellularly with those previously obtained intracellularly to identical visual stimuli. Remarkably, the classifications made by this criterion rendered groups of units that could be distinguished by criteria different from the one originally used to separate them. The units gathered as BLG2 have a waveform that allows to distinguish them from units grouped as MLG2 and LCDC. Likewise, an analysis of the auto-correlograms allows us to separate the units of the three groups. The similarity in the waveforms and the auto-correlograms found among units that were ascribed to each one of the three groups based solely on their response profile, and the differences between the units of the separated groups, provide strong validation on our criteria of spike sorting and of neuronal identification.

The highest firing rates reached with extracellular recordings were a bit lower than those obtained with intracellular recordings (Figure 4). This may be because looming stimuli are known to induce high frequency firing with similar latency in different LG neurons, especially towards the end of image expansion (Tomsic et al., 2017). Hence, regularly occurring overlapping spikes of different neurons may be interpreted as separate waveforms of a particular neuron. Also, false-negative errors may include misclassification because of a reduction in amplitude and an increase in width for the trailing spikes of a burst. Consequently, at high firing frequency, the spike waveforms of an individual unit become irregular and may not be recognized by the software to be included in the cell cluster (Bhavsar et al., 2015).

The two previously characterized LG classes named MLG1 and BLG1 have been elusive in our experiments. Several reasons may account for their lack. First, both classes are composed of several units whose anatomical and physiological receptive fields are considerably smaller compared to those of the MLG2 and BLG2 classes, which are thought to be represented by one single element per lobula (Medan et al., 2007). In our experiments, the stimulation area was restricted to the screen location, which encompassed a small portion (less than 25%) of the horizontal visual space seen by the crab’s eye. This, in combination with the receptive field size of MLG1 and BLG1, could have made these neurons less likely to be activated. Another reason would be related to a low level of spontaneous activity, in particular for MLG1 neurons. Because the reliability of clusters formation depends on the number of detected spikes, neurons with high spontaneous activity are better isolated than neurons that are only activated by the presence of stimuli. In our experiments, the sum of time corresponding to the presentations of all the stimuli comprised less than 4% of the entire duration of the recording. Therefore, for neurons like MLG1s that barely show activity in absence of stimulation, the effectiveness of building reliable clusters is compromised. This being said, we have recorded two units whose response profiles resemble that of MLG1 neurons, but their endorsement is pending until more similar units will be recorded.

### Unidentified Recorded Units

Although the present study is focused on the identification of neurons previously characterized by intracellular recordings, a brief discussion on the unidentified units is warranted. Most of the recorded units exhibited response profiles distinct to those that characterize the particular classes of LG neurons or the LCDCs. This is not surprising given that the neurons so far characterized likely represent a fraction of the large tangential elements present in the lobula. Indeed, while attempting to record intracellularly from LG neurons, we often impale neurons that display differential sensitivities for particular visual stimuli. We have not systematically studied these neurons yet. However, comparable response preferences could be observed in some of our extracellularly recorded units. For example, a unit responded with excitation to the large moving square and with inhibition to the small square; another unit responded with similar excitation to all square sizes followed by marked post-stimulus suppression of the spontaneous activity; a unit displayed a stronger response to the grating pattern when it was presented motionless than when it moved; several units responded with transient excitations at the beginning and the end of the square translatory motion. A thorough description of these types of units is pending on further studies.

### Functional Neuronal Interactions

Transformation, transmission, and storage of information in the brain are achieved by the cooperative action of neuronal ensembles. The study of population activity of neurons in the crab has been satisfied so far by artificially combining data obtained through intracellular recordings from different individuals (Tomsic et al., 2003; Sztarker and Tomsic, 2011; Oliva and Tomsic, 2014, 2016; Medan et al., 2015). There have been double intracellular recordings performed to study combined responses of different neurons (Scarano et al., 2018), but the success rates for simultaneous recordings in the living crab is usually quite low. Population neural responses have also been studied in the crab by using massive staining and optical recording (Berón de Astrada et al., 2013), however, this methodology does not allow revealing the identity of individual units. Besides, while optical recording methods provide the advantage of spatial information, their temporal resolution does not meet the requirement for assessing the information encoded in the high firing frequency used by neurons (Brill et al., 2013). Thus, simultaneous access to single neurons in the same preparation at high temporal resolution can only be achieved through extracellular multichannel recording. By analyzing the temporal relationship of activity between simultaneously recorded units using cross-correlograms, it is possible to infer different kinds of neuronal interactions (e.g., Barthó et al., 2004; Hill et al., 2011). Nevertheless, the number of detectable interactions is usually low (e.g., Barthó et al., 2004). We recorded different types of interactions. For instance, a likely monosynaptic (short-latency, sharp peak) excitatory synaptic connections of BLG2 on an unidentified unit (Figure 6A), a likely mono or disynaptic (<10 ms delay) inhibitory connection of an unidentified unit on the BLG2 (Figure 6B), and a reciprocal interaction involving more complex functional relations between the MLG2 and the BLG2 neurons (Figure 6C). The connection between this pair entails a short-latency and long-lasting excitatory effect of the MLG2 on the BLG2 and an indirect (>10 ms delay) inhibitory effect of the BLG2 on the MLG2. An inhibitory connection of the BLG2 on the MLG2, such as the one observed here, has been anticipated by the analyses of the temporal course of response of these neurons to a variety of looming stimuli. Moreover, the interaction has been proposed to be part of the neural mechanism underlying the decision of switching from a freezing response to an escape response (Oliva, 2010). When a crab faces an approaching object, its first strategy is to freeze, but if the object continues to approach the crab runs away. The BLG2 neuron strongly responds to a looming stimulus at the very beginning of its expansion, when the freezing occurs (Tomsic et al., 2017). Thus, the activity of the BLG2 may lead to freezing while contributing to inhibit the MLG2. If the stimulus further approaches the activity of the BLG2 decays, releasing the MLG2 that starts firing and the crab begins to run away (Oliva, 2010). Once the escape has been launched, the response of the MLG2 neuron faithfully encodes the angular velocity of looming stimuli, and thus conveys the information used by the animal to continuously adjust its running speed (Oliva and Tomsic, 2016).

### Toward Simultaneous Multiunit Recording in the Freely Moving Crab

Field and laboratory studies have demonstrated that the crab’s avoidance behavior is not a stereotyped reflex reaction, but a complex repertoire of strategies that includes freezing, escaping, and confronting. The decision on which strategy should be implemented is based on risk assessment, for which the animal takes into account the stimulus as well as the contextual situation, such as the availability of a near shelter (Hemmi and Tomsic, 2012). When running away from a visual threat the crab continuously adjusts its direction and speed of escape according to changes in the incoming visual information (Oliva and Tomsic, 2016; Medan et al., 2015). Besides, the escape response to a specific stimulus can be rapidly adapted by learning (Tomsic and Maldonado, 2013). By recording intracellularly from immobilized animals we have shown that some of these behavioral attributes are reflected by the activity of the LG neurons (e.g., Sztarker and Tomsic, 2011; Oliva and Tomsic, 2014, 2016; Medan et al., 2015), which lead us to propose that these neurons form a microcircuit that acts as a decision-making node (Tomsic, 2016). The correspondence of the activity of particular LG neurons with a distinct component of the escape response was established by the remarkable matching found between the temporal course of the neuronal and the behavioral responses to a variety of visual stimuli measured separately in different individuals. However, the neural control of elaborated behaviors can hardly be understood by the analysis of single-neuron physiology. Simultaneously recording the individual activity of the foremost neurons of the circuit involved in the avoidance responses of the crab will considerably improve our knowledge on the neural interactions and computations underlying the organization of these behaviors. This goal became more realistic after having confirmed, as we did here, that the identity of LG neurons can be faithfully recognized from extracellular recorded units.

The stability of our recordings in combination with the suitable size and robustness of the crab gives us confidence in the feasibility of recording from the freely moving animal. Besides, the readiness of the crab to behave in the laboratory, where stimulation conditions are well controlled and responses are easy to measure, offers excellent opportunities for evaluating the conjoin activity of lobula neurons in the behaving animal.

## Data Availability Statement

All datasets presented in this study are included in the article.

## Ethics Statement

The research was conducted in accordance with the Ethical Reference Frame for Biomedical Investigations of the Consejo Nacional de Investigaciones Científicas y Técnicas de Argentina, equivalent to the standard procedures for animal care and use of the US NIH.

## Author Contributions

All authors had full access to all the data in the study and take responsibility for the integrity of the data and the accuracy of the data analysis. DT helped in study concept and design, helped in drafting of the article, and obtained funding. AC performed the experiments and acquired the data. AC, MB, and DT helped in analysis and interpretation of data, and helped in critical revision of the article. All authors contributed to the article and approved the submitted version.

## Conflict of Interest

The authors declare that the research was conducted in the absence of any commercial or financial relationships that could be construed as a potential conflict of interest. The reviewer NM declared a shared affiliation, with no collaboration, with the authors to the handling editor at the time of review.

## Footnotes

^1^http://klustakwik.sourceforge.net
